# How Does Inclusive Leadership Curb Workers’ Emotional Exhaustion? The Mediation of Caring Ethical Climate and Psychological Safety

**DOI:** 10.3389/fpsyg.2022.877725

**Published:** 2022-07-07

**Authors:** Xintian Li, Peng Peng

**Affiliations:** ^1^School of Business, Shandong Jianzhu University, Jinan, China; ^2^School of Business, Qingdao University of Technology, Qingdao, China

**Keywords:** inclusive leadership, psychological safety, caring ethical climate, emotional exhaustion, hospitality frontline employees

## Abstract

The COVID-19 pandemic has transformed the politics, economy, and society of the world, which has dealt the most severe blow to the hospitality industry. Meanwhile, the pandemic and government control policies have brought high psychological pressure to hospitality front-line employees, resulting in emotional exhaustion. As a part of burnout syndrome, emotional exhaustion poses a threat to employees’ mental health, career sustainability, and well-being. Therefore, the purpose of this paper was to investigate the curb effectiveness of inclusive leadership on emotional exhaustion and to explore the mediation roles of ethical climate and psychological safety between them. Time-lagged data were collected from 65 teams and 358 hospitality front-line employees working in Chinese hotels in two stages with a one-month gap. This research verified that inclusive leadership has a negative impact on emotional exhaustion both indirectly through the mediation roles of ethical climate and psychological safety. And the ethical climate and psychological safety played partial mediation roles between inclusive leadership and emotional exhaustion. In theory, the findings explored the dual mediation mechanism of the inhibitory effect of inclusive leadership on emotional exhaustion. In practice, we provided the training and correct guidance to develop inclusive leadership for hotel enterprises and to resolve the emotional exhaustion of employees, which can enhance sustainability in careers.

## Introduction

With the COVID-19 pandemic spreading at a rapid rate since 2019, the global economy must face leaner times, especially in the hospitality industry (Chen et al., 2020a). The hospitality industry is a labor-intensive industry, and the exploitation and management of its human resources could become the key element to acquiring the competitiveness of hotels. The hospitality front-line employees need to face customers directly to fulfill customers’ diverse demands and to satisfy their individualized requirements of customers. At this time, personality traits of customers (such as irritability, sensitivity, suspicion, etc.), language communication methods (such as forceful, impolite, swear words, etc.) will directly put pressure on hotel employees which leads them easy to get frustrated with customers’ complaints. On the other hand, the pandemic has significantly changed the work-non-work interface, increasing work stress and emotional exhaustion for front-line employees (Choi et al., 2014; Han et al., 2016; Vaziri et al., 2020).

The COVID-19 pandemic has heightened uncertainty about the future of the hospitality industry and increased the psychological insecurity of employees which affected their psychological and work consequences during the unemployment or discontinuous employment period (Zhao et al., 2020; Liu et al., 2021). Psychological safety is an important psychological state that can affect individual work engagement. Managers usually expect their employees should be enthusiastic, creative, and responsible in performing their duties, but employees usually fail to meet such expectations. Psychological safety is a feeling of confidence, security and freedom from fear and anxiety at work (Witte, 1999). Individual safety evaluation of work role affects job performance by influencing attitude and perception of work. Previous studies found that psychological insecurity is a source of job stress which led to negative job results. Such as reduced job satisfaction, job engagement and performance, and even increased emotional exhaustion (Schumacher et al., 2016; Akgunduz and Eryilmaz, 2018; Chen et al., 2020b). Once employees enter a state of exhaustion, there will be a series of physical and mental reactions. Such as physical and mental fatigue, work efficiency decreases, numbness, emotional control ability decreases, and even occurs interpersonal conflict. Finally, they may lose confidence in themselves and their work, become depressed and withdrawn, and hinder their sustainable career development.

Researchers have explored the variables that curb workplace emotional exhaustion, most directly by taking time off from the source of stress to rest and replenish the emotional “pool.” In addition, personal identity, managers, and social support can help employees manage their work emotions, empower employees, and create greater value for the enterprise (Reichl et al., 2014; Langley et al., 2019). In the meantime, social support and a caring ethical climate can help to reduce the emotional exhaustion of the front-line employees (Karatepe and Kilic, 2015). Inclusive leaders can help employees follow the company’s vision, manage their behavior, and rise above emotional exhaustion (Asif et al., 2019; Yang et al., 2019), which is recognized as the most appropriate leadership behavior in today’s challenging environment (Javed et al., 2018). When an inclusive leader shows an inclusive attitude towards employees’ work mistakes, it will encourage employees to boldly try new work methods when solving problems, thus improving employees’ work competence and promoting their positive behaviors (Shore et al., 2011). Therefore, inclusive leadership can increase employees’ willingness to take chances and promote positive behaviors (Hollander, 2009). It also addresses employees’ need to belong, helping them curb emotional exhaustion and improving their career sustainability (Randel et al., 2018). In conclusion, inclusive leadership could enhance employees’ sense of belonging and responsibility, making them suitable for suppressing emotional exhaustion through inclusion, employee participation, and risk sharing (Hollander, 2009; Shore et al., 2011).

Although much of the previous research has focused on the importance of leadership and emotional exhaustion, insufficient attention has been paid to the inhibitory mechanisms of inclusive leadership on emotional exhaustion. Inclusive leadership can make employees experience the feelings of competency, autonomy, and tolerance in their jobs, and may decrease the emotional exhaustion of employees. The influence of leadership on employee behavior is often indirect (Qi and Liu, 2017), so it is necessary to examine the mediation mechanisms of the relationship between inclusive leadership and emotional exhaustion. The first purpose of this study was to examine the impact of inclusive leadership on emotional exhaustion. To confirm that we rely on the COR theory to explain the impact of inclusive leadership on emotional exhaustion. The second purpose was to examine the mediating roles of caring ethical climate (organizational climate) and psychological safety (individual psychological characteristics) between inclusive leadership and emotional exhaustion because they can help reduce emotional exhaustion at work. Thirdly, this study tries to establish a theoretical framework and inhibition mechanism for inclusive leadership and emotional exhaustion. Eventually constructed a dual mediation model of inclusive leadership, psychological safety, a caring ethical climate, and emotional exhaustion. The results of the present study can help clarify the factors that inhibit emotional exhaustion among front-line hotel employees and provide insights into HRM for managers in potential crises. This study also fills a key gap in the literature by explaining how inclusive leadership affects emotional exhaustion in this context.

## Literature Review and Hypotheses Development

### The Relationship Between Inclusive Leadership and Emotional Exhaustion

Inclusive leadership was first proposed in the field of organizational management, aiming to solve the problems caused by employee diversity. It is embodied in paying attention to the needs of subordinates and demonstrating openness, effectiveness, and accessibility in their interactions with subordinates (Nembhard and Edmondson, 2006). Subsequently, Carmeli et al. (2010) proposed inclusive leaders are those who demonstrate visibility, accessibility, and availability when interacting with subordinates. Hollander (2009) discussed inclusive leadership from the perspective of the interdependent relationship between leaders and employees, emphasizing mutually beneficial relationships and shared visions. Scholars have explored the effectiveness of inclusive leadership; however, these studies have mainly focused on the individual level, such as adaptability, performance, and engagement of employees (Hirak et al., 2012; Choi et al., 2014, 2017; Randel et al., 2018). In addition, the existing literature studies the relationship among inclusive leadership and psychological safety (Kim et al., 2018), employees’ psychological capital and innovative work behavior (Javed et al., 2017), strengthens the employee s’ sense of belonging (Randel et al., 2018), improve the employees’ creativity (Zhu et al., 2019). Recently, the relationship between inclusive leadership and individual motivation level has been a hot research topic in management research (Di Fabio and Peiró, 2018), but there are relatively few in-depth discussions on the influence mechanism of inclusive leadership on employee negative behavior.

Burnout refers to a multidimensional concept, including emotional exhaustion, depersonalization, and reduced personal accomplishment (Maslach and Jackson, 1981). Emotional exhaustion is the central dimension of burnout, which is mainly manifested as fatigue and the sense of burnout caused by work (Maslach and Jackson, 1981; Wright and Cropanzano, 1998). This happens when a person is emotionally overwhelmed and physically exhausted by extreme work or personal demands (Maslach and Leiter, 2008). It manifests itself as physical fatigue, as well as a feeling of mental and emotional exhaustion. Emotional exhaustion is most likely to occur when there is an actual loss of resources, a perceived threat of loss of resources, or there are not enough resources to meet the needs of the work, the resource investments did not pay off as expected (Hobfoll, 1989). Emotional exhaustion also occurs when individuals feel that they no longer have sufficient emotional resources to deal with the stressors (Hobfoll, 1989; Lee and Ashforth, 1996). With the increase in work requirements, the workload of front-line hotel staff is gradually increasing, and the surrounding environment brings great pressure on them. So that these complex situations will bring negative psychological and work consequences to employees, resulting in emotional exhaustion (Zhao et al., 2020; Liu et al., 2021; Zhao and Jiang, 2021). The cost of emotional exhaustion is reduced job satisfaction, organizational commitment, job performance, job performance voluntary turnover, diminished job performance, and an increased level of turnover intention (Rathi and Lee, 2016). Emotionally exhausted people have higher levels of work-life conflict. The long-term drain on resources can even have a serious impact on mental health and well-being. Although emotional exhaustion can occur in any profession, hospitality front-line employees are more prone to burnout as they need to face customers directly and meet their emotional and material needs with limited work resources (Bakker and Demerouti, 2007). During the COVID-19 pandemic, restaurant front-line employees need to devote more physical and mental resources simultaneously to cope with work demands, which may lead to excessive loss of employees’ resources and emotional exhaustion. Therefore, how to curb the emotional exhaustion of hospitality front-line staff in such a critical situation is particularly important and worth further discussion.

The study suggested that the Conservation of Resource (COR) theory may help to understand the relationship between inclusive leadership and emotional exhaustion of hospitality front-line employees (Hobfoll, 1989). Hobfoll (1989) defined resources as “objects, personal characteristics, conditions, or energies cherished by individuals or used as means to achieve these goals.” And anything of value to someone can be considered a resource (Thompson and Cooper, 2001; Gorgievski et al., 2011). COR theory begins with the belief that individuals strive to acquire, retain, nurture, and protect the things they hold dear. COR theory has been adopted in many areas of the stress spectrum, from burnout to traumatic stress (Hobfoll et al., 2018). According to the basic concept of COR theory, individuals strive to conserve their existing resources (conservation) and acquire new resources (acquisition; Hobfoll, 2004), thereby reducing the loss of any resources. Emotional exhaustion occurs when people perceive the potential threat of resource loss, actually lose the resource, and can no longer obtain the resource after investment (Hobfoll, 1989).

The job resources (such as knowledge, emotions, and relationship resources) provided by inclusive leaders help employees achieve work goals, reduce job stress, prevent physical and mental exhaustion, and promote personal performance and well-being (Demerouti et al., 2001). Inclusive leaders are good at listening and respecting employees, reflecting the way of thinking and behavior of “tolerance is greatness” and “harmony without difference.” Inclusive leadership can provide resources for the resource consumption of team employees and enhance the positive emotions of employees. Relevant studies confirmed that inclusive leadership can significantly improve employee initiative, innovative work behavior, and voice (Javed et al., 2018; Lee and Dahinten, 2021). Inclusive leaders play an important role in restraining employees’ psychological stress by providing them with a relaxed and reassuring working environment (Zhao et al., 2020). The above research showed that the supportive behavior of inclusive leaders can reduce employees’ uncertainty, anxiety, and work pressure, and restrain emotional exhaustion. Therefore, we propose the following hypothesis:

*H1*: Inclusive leadership has a negative relationship with emotional exhaustion of employees.

### The Mediating Role of Caring Ethical Climate

Victor and Cullen (1988) referred to five dimensions of ethical climate (law and norms, caring, independence, instrumentalism, and rules) in their research. Caring ethical climate is an important part of the organizational atmosphere which mainly refers to the common cognition of employees on policies, procedures, and systems within the organization and influences employees’ behaviors by emphasizing friendship and team interest (Cullen et al., 2003). In a caring climate, the primary consideration is what is best for everyone in the organization. In a specific organizational environment, an ethical climate can correctly guide the moral attitude, beliefs, and motivation of employees.

Previous studies have explored the causes and consequences of ethical climate and believe that leadership has a greater and more ethical impact on ethical climate than other variables. Because employees take the leader’s behavior as a reference when the leader’s behavior is more ethical, the subordinate employees’ behavior is also more ethical (Calabrese and Roberts, 2002; Rathert and Fleming, 2008). These studies provided insights into dominance and social salience and demonstrated the importance of inclusive leadership in improving the ethical climate.

According to ethical climate theory, providing an ethical climate in terms of norms, rules, culture, and policies in an organization can reduce an individual’s level of negativity. A positive organizational climate can act as a resource that can reduce the impact of a resource loss situation (Hobfoll, 1989). Previous studies on outcome variables of ethical climate mainly focus on individual psychological, behavior, innovation, and incentive productivity (Huang et al., 2012; Qi and Liu, 2017). Dollard and Bakker (2010) proposed that a positive organizational climate leads to low demands and high resources, which in turn protects employees from adverse health outcomes such as burnout, anxiety, and fatigue. A caring ethical climate may act as a buffer, reducing the harmful effects of work requirements and protecting employees from hazardous situations (Loh et al., 2018). Joe et al. (2018) demonstrated that a strong perceived caring climate reduces emotional fatigue and job dissatisfaction of employees which caused turnover intention. Hence, a positive caring ethical climate can act as a ‘resource caravan’, encouraging the formation of a pool of resources that will help reduce the risk of burnout (Hobfoll, 2004).

In conclusion, according to the COR theory, we propose that inclusive leadership can form a caring ethical climate in a team, thus inhibiting the occurrence of emotional exhaustion. Therefore, the following hypothesis is proposed.

*H2*: Inclusive leadership has a positive relationship with caring ethical climate.

*H3*: Caring ethical climate has a negative relationship with emotional exhaustion of employees.

*H4*: Caring ethical climate exerts mediating effect on inclusive leadership and emotional exhaustion of employees.

### The Mediating Role of Psychological Safety

Psychological safety means that you feel safe interacting with others without fear that you will be embarrassed or punished in some way. While facing the problems in the organization, employees who are fearless and put forward their opinions, concerns, and unique ideas for improvement regardless of interpersonal relationships and occupational risks. The psychological contract is a kind of expectation of the labor-management relationship outside the formal contract (Keim et al., 2014). Inclusive leadership increases employees’ expectations of assistance from their superiors when needed and enhances employees’ work vitality and self-efficacy (Kurtessis et al., 2017). Currently, when employees receive support from their superiors, the organization, and the surrounding environment, they tend to show better job participation, such as a higher level of work vitality, a more focused work style, and more absorption of work tasks (Leiter and Bakker, 2010). In addition, Labrague and De Los Santos (2020) also found that psychological safety can reduce psychological distress and career turnover intention of front-line employees.

The inclusive nature of openness, availability, and accessibility in the workplace enables leaders to help employees more easily perceive psychological safety (Detert and Burris, 2007). Therefore, inclusive leaders value and integrate employees into specific work processes, giving employees more opportunities to put forward their own opinions on work and organization, then generate, promote, and implement these useful ideas (Hirak et al., 2012; Boekhorst, 2015). Such inclusive leaders are more conducive to helping enterprises or organizations form an organizational culture in which employees’ ideas and opinions are highly valued and respected by superiors. These inclusive leaders care about the feelings and expectations of their employees. As a result, employees are more likely to feel psychological safety when working with supportive and inclusive leaders in stressful situations (Carmeli et al., 2010; Detert and Edmondson, 2011). At this point, the openness shown by inclusive leaders is more likely to encourage employees to take innovative actions and ensure that employees will not be punished when negative results occur so that employees can obtain greater psychological safety (Carmeli et al., 2010). Inclusive leaders are good at encouraging employees to contribute their ideas in direct cooperation with employees and helping them develop psychological safety (Nembhard and Edmondson, 2006). Inclusive leadership focuses on open communication and building strong relationships with employees who feel psychologically safe enough to take risks that may arise in their work (Carmeli et al., 2009; Shore et al., 2011). Based on those research results (Nembhard and Edmondson, 2006; Carmeli et al., 2010; Hirak et al., 2012; Javed et al., 2017; Ye et al., 2019), a positive correlation between inclusive leadership and psychological safety can be inferred.

Psychological safety can also help supervisors and employees to establish better relations based on mutual respect and help employees to perceive support from others in the organization. Psychological safety strengthens self-awareness, regulates emotional responses, and avoids emotional exhaustion (Nembhard and Edmondson, 2006). At the same time, previous studies have confirmed that psychological safety is the mediation between inclusive leadership and change-oriented behavior (Carmeli et al., 2010; Javed et al., 2017). Therefore, we hypothesized that psychological safety could mediate the relationship between inclusive leadership and emotional exhaustion. We propose the following hypothesis:

*H5*: Inclusive leadership has a positive relationship with psychological safety.

*H6*: Psychological safety has a negative relationship with emotional exhaustion of employees.

*H7*: Psychological safety exerts mediating effect on inclusive leadership and emotional exhaustion of employees.

## Materials and Methods

### Sample and Process

The hotel and catering industry is labor-intensive, and the service industry is more concerned about emotional exhaustion than the production industry during the COVID-19 pandemic. The study sample included hospitality front-line employees aged 18 and above in China. We purposefully selected these respondents from the hospitality industry based on the following considerations. First, hospitality front-line employees are more likely to experience high levels of emotional exhaustion in their daily work during the COVID-19 pandemic (Han et al., 2016). Second, through interviews with these hotel managers, we found that emotional exhaustion among front-line employees has increased recently. With the help of the HR department, online self-report surveys were conducted *via* Wechat (SNS application) and email, respectively. We informed the respondents about the purpose of this research and ensured that information was kept completely confidential. To avoid common methodological bias, questionnaires were collected in two different stages (Li and Tangirala, 2021).

We measured independent variables (inclusive leadership), mediator variables (psychological safety and caring ethical climate), and control variables using survey 1 in the time 1 phase during 1 week from December 15th, 2021. Survey 1 consisted of 23 items, and a total of 423 responses from 65 teams were returned at this stage. One month later, we measured the dependent variable (emotional exhaustion) of the same participants, using survey 2 which included 9 items in the time 2 phase. A total of 363 responses were matched at time 2 and time 1. After screening for incomplete answers and missing values, the final sample included 358 employees from 65 teams for data analysis, with an effective recovery rate of 84.6%.

### Measures

All relevant contents of the questionnaire are translated into Chinese according to the translation-back Translation Procedure (Brislin, 1970). The answer to the questionnaire was designed to utilize five-point Likert Scale (1 = strongly disagree, 5 = strongly agree).

#### Inclusive Leadership Scale

We used a three-dimensional, 9-item scale developed by Carmeli et al. (2010) to measure inclusive leadership. Specific questions included “The manager is always willing to listen to others’ new ideas” and “The manager encourages me to contact him/her about emerging problems.” The Cronbach’s coefficient of the inclusive leadership scale was 0.887 in this study.

#### Psychological Safety Scale

We used a three-item PS scale developed by May et al. (2004) to measure psychological safety. Specific questions included “I am not afraid to be myself at work,” “I am afraid to express my opinions at work,” and “There is a threatening environment at work.” These projects assess whether individuals are willing to express their opinions even in a threatening work environment. The Cronbach’s coefficient of psychological safety scale was 0.896 in this study.

#### Caring Ethical Climate

We measured caring ethical climates with 7 items based on Ethical Climate Questionnaire Scale developed by Victor and Cullen (1988). Specific questions included “The most important of these is the overall benefit of the team members,” “Our focus is usually on what’s best for the other members in the team,” and “In the whole team, members take good care of each other.” The Cronbach’s coefficient of caring ethical climate was 0.867 in this study.

#### Emotional Exhaustion

We measured emotional exhaustion with 5 questions based on the emotional exhaustion dimension of the Chinese Maslach Burnout Inventory (Li et al., 2005). The Maslach Burnout Scale (MBI) is a commonly used scale to assess burnout levels in individuals (Maslach and Jackson, 1981). The scale has been applied to many occupations in China and both reliability and validity met the needs of psychometric standards (Li and Tan, 2009). More specifically, our study provided the Cronbach’s coefficient of emotional exhaustion was 0.905 by using five items which included “I am very tired,” “I often feel exhausted,” “I am knackered after working all day,” “I am afraid the work will affect my mood” and “I feel a little depressed.”

#### Control Variables

In this study, we used four demographic variables: gender, age, education level, and organizational tenure. Gender was measured using a dummy variable (0 for female and 1 for male). Age was measured in years and education levels were measured on a scale ranging from 1 to 4. Organizational tenure was measured by self-describing years of service with the organization.

### Methodology

First, we used SPSS 22.0 for descriptive analysis, reliability analysis, and correlation analysis. Second, we examined the unique validity of current variables in this paper through confirmatory factor analysis (CFA). The variables used in this paper include inclusive leadership, emotional exhaustion, caring ethical climate, and psychological safety. Third, we estimated path coefficients and three-path indirect effects as well as 95% bootleg confidence intervals (CI), using methods recommended by Shrout and Bolger (2002) and Preacher and Hayes (2004). Yet bootstrapping is more advantageous than a normal distribution-based significance test (Shrout and Bolger, 2002).

## Results

### Descriptive Statistics

Table 1 showed the demographics of the respondents: 207 (57.8%) females and 151 (42.2%) males. In terms of age, the respondents are below 20 years old (8.7%), 21–30 years old (71.2%), 31–40 years old (10.7%), and over 40 years old (9.4%). Most respondents are between 21 and 30 years old (71.2%), which reflects that the service personnel in China’s hospitality are all relatively young. In terms of education, 25.8 percent were high school or lower, 42.1 percent were junior college, 23.8 percent were bachelor’s, and 8.3 percent were masters. The average tenure is 2.5 years. Our study found that there was a significant correlation between inclusive leadership and psychological safety (*r* = 0.43, *p* < 0.01), inclusive leadership and caring ethical climate (*r* = 0.47, *p* < 0.01), inclusive leadership and emotional exhaustion (*r* = −0.41, *p* < 0.01), psychological safety and emotional exhaustion (*r* = −0.51, *p* < 0.01), caring ethical climate and emotional exhaustion (*r* = −0.45, *p* < 0.01). Our preliminary results confirmed the previous research hypothesis in this paper.

**Table 1:** Descriptive statistics and correlations for the study variables.

**S. No.**	**Variable**	**Mean**	**SD**	**AVE**	**CR**	**1**	**2**	**3**	**4**	**5**	**6**	**7**	**8**
1.	Gender	0.57	0.68	–	–	1							
2.	Age	26.55	7.92	–	–	0.08	1						
3.	Educational level	2.52	0.61	–	–	0.11	0.10	1					
4.	Organizational tenure	2.76	0.92	–	–	0.09	0.21	0.07	1				
5.	Inclusive Leadership	3.89	0.63	0.62	0.91	0.17	0.18	−0.12	0.31	1			
6.	Psychological Safety	3.92	0.57	0.67	0.93	0.09	0.02	0.31	0.03	0.43^**^	1		
7.	Caring Ethical Climate	3.27	0.59	0.63	0.81	0.07	0.0	0.07	0.15	0.47^**^	0.21^**^	1	
8.	Emotional Exhaustion	3.95	0.62	0.61	0.87	0.18	0.02	−0.67	0.29	−0.41^**^	−0.51^**^	−0.45^**^	1

Gender (0 = female, 1 = male), age (1 = less than 20, 2 = 21–30, 3 = 31–40, 4 = more than 40), educational level (1 = high school and below 2 = college, 3 = bachelor’s, 4 = master’s); *N* = 358.

** *p* < 0.01.

### Composite Reliability and Average Variance Extracted

In the present paper, average variance extraction (AVE) and compound reliability (CR) were evaluated to confirm convergence validity and discriminant validity (Fornell and Larcker, 1981). If the AVE value is greater than or equal to 0.50 while the CR value is greater than or equal to 0.60 (Bagozzi and Yi, 1988), the convergence validity can be determined. The research results in Table 1 showed that convergence validity has been established in this study as the AVE value of the four potential variables in this study decreased from 0.61 to 0.67 and the CR value decreased from 0.81 to 0.93. Next, if the square root of the AVE of a structure exceeds the correlation of the structure with other structures in the model, then the validity of the distinction in the study can be ensured. Table 1 of this paper showed that the square root of the AVE of each structure exceeded the correlation level between the structure and other structures in the model, confirming the discriminant validity of the study.

### Confirmatory Factor Analysis

This paper used confirmatory factor analysis (CFA) to validate the measurement model before testing the hypothesis (Anderson and Gerbing, 1988). The measurement model consists of four potential factors: inclusive leadership, psychological safety, caring ethical climate, and emotional exhaustion. The CFA results of this study are shown in Table 2. The data of the 4-factor model were in good fit [*χ*^2^ (209) = 325.384, values of CFI = 0.917, TLI = 0.921, and RMSEA = 0.052]. This proved that the goodness of fit of the model is significantly better than other factor models (three-factor model, two-factor model, and single-factor model), indicating that the measurement has good discriminant validity. The results showed that the measurement model fits well with the data.

**Table 2:** Confirmatory factor analyses results.

Model	*χ* ^2^	df	*χ*^2^/df	CFI	TLI	RMSEA
Four-factor model^a^	325.384	209	1.557	0.917	0.921	0.052
Three-factor model^b^	453.872	212	2.141	0.877	0.882	0.069
Three-factor model^c^	467.121	212	2.203	0.869	0.875	0.072
Two-factor model^d^	565.727	214	2.643	0.785	0.795	0.095
Single factor model^e^	675.917	215	3.144	0.691	0.617	0.159

a In the four models, there is no relationship between all variables measured.

b Merging inclusive leadership and caring ethical climate into a potential factor.

c Merging inclusive leadership and psychological safety into a potential factor.

d Merging inclusive leadership, psychological safety, and caring ethical climate into a potential factor.

e Merging all variables into a potential factor.

### Common Method Variance

Measurement errors may occur in commonly used variance variables because the common method variance is related to the measurement method rather than the structure of the measurement item itself (Williams and Anderson, 1994; Podsakoff et al., 2003). Therefore, this study used two methods to reduce the common variance. First, we collected the questionnaire through two different stages, and this time difference would reduce the effect of common variance due to the same continuity scale (Podsakoff et al., 2003). Second, we used Harman’s single factor to test whether the studies had common method variances (Podsakoff et al., 2003). The exploratory factor analysis results of this study showed that the first factor could only explain 43.214% of the variance which is lower than the threshold of 50% (Shiau and Luo, 2012). So, there is no significant common method variance was observed in the study, and the data analysis results were within the acceptable range.

### Measurement Model

This study uses structural equation modeling (SEM; Anderson and Gerbing, 1988) to test the proposed hypothesis. Before verifying the hypothesis, one-way ANOVA was first used to determine whether demographic variables had an impact on emotional exhaustion of employees. However, data analysis results showed that demographic data did not have a significant impact on emotional exhaustion. Therefore, we excluded demographic variables from the hypothesis of this study.

**Figure 1:**
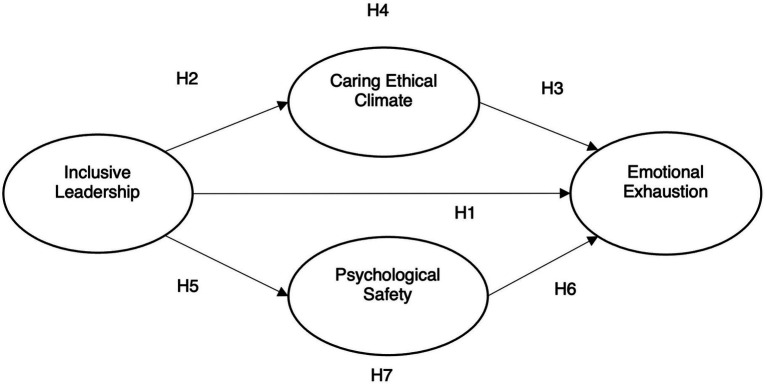
Analysis model. ^*^p < 0.05; ^**^p < 0.01; ^***^p < 0.001. *χ*^2^ = 378.652 (df = 223); *χ*^2^/df = 1.698, GFI = 0.903, NFI = 0.924, RMSEA = 0.043, TLI = 0.927, CFI = 0.932.

The path diagram of the structural model in this paper is shown in Figure 1. The data analysis results showed that *χ*^2^/DF = 1.698, GFI =0.903, CFI =0.932, NFI =0.924, TLI =0.927, and RMSEA =0.043, all the related indexes were in line with the rationality model standard. It can be shown that the model of this study fits well (Hair et al., 2012). According to the research results shown in Table 3. Hypothesis 1, the negative effects of inclusive leadership on emotional exhaustion were examined and accepted (*β* = −0.159, *p* < 0.01). Similarly, hypothesis 2, inclusive leadership was significantly positively correlated with the ethical climate of caring, and the outcome was accepted (*β* = 0.221, *p* < 0.001). Hypothesis 3 examined the negative effects of caring ethical climate on emotional exhaustion and the outcome was supported (*β* = − 0.115, *p* < 0.05). Hypothesis 5 examined the positive effect of inclusive leadership on psychological safety and the outcome was accepted (*β* = 0.207, *p* < 0.001). Finally, hypothesis 6 examined the negative effects of psychological safety on emotional exhaustion and the outcome was also supported (*β* = −0.197, *p* < 0.01).

**Table 3:** The path analysis results.

Paths			
Direct effects	Path coefficient	*t*-value	Result
Inclusive leadership →emotional exhaustion (H1)	−0.159^**^	3.120	Accept
Inclusive leadership →caring ethical climate (H2)	0.221^***^	4.652	Accept
caring ethical climate →emotional exhaustion (H3)	−0.115^*^	1.979	Accept
Inclusive leadership →psychological safety (H5)	0.207^**^	4.327	Accept
Psychological safety →emotional exhaustion (H6)	−0.197^**^	2.675	Accept

*p < 0.05; **p < 0.01; ***p < 0.001.

Hypothesis 4 and 7 suggested that both the caring ethical climate and psychological safety promote the correlation between inclusive leadership and emotional exhaustion. To test the mediating effect in this paper, we conducted a bootstrap test with AMOS with 5,000 bootstrap samples. The study followed the procedure outlined by Preacher and Hayes (2008) in two separate mediation analyses. The data in Table 4 showed that caring ethical climate significantly mediated the relationship between inclusive leadership and emotional depletion (*β* = 0.131, *p* < 0.01, 95% bias-corrected confidence intervals [95% CI] ranging from 0.062 to 0.184). Psychological safety was also found to mediate the relationship between inclusive leadership and emotional exhaustion (*β* = 0.172, *p* < 0.001, 95% CI [0.025 to.138]). The study concluded that both caring ethical climate and psychological safety partially mediated the relationship between inclusive leadership and emotional exhaustion. The result of this study is consistent with Zhao et al. (2010) research on the mediating effect of caring ethical climate and psychological safety. Therefore, hypothesis 4 and hypothesis 7 of this study have been verified.

**Table 4:** The mediating effect of the model.

Mediating effects	Indirect	BC (95% CI)	Degree of mediation
Inclusive leadership →caring ethical climate →emotional exhaustion (H4)	0.131^**^	[0.062, 0.184]	Partial mediation
Inclusive leadership →psychological safety →emotional exhaustion (H7)	0.172^**^	[0.025, 0.138]	Partial mediation

**p < 0.01.

## Discussion

### Theoretical Significance

Based on COR theory, this study extended the effectiveness of inclusive leadership to emotional exhaustion in service settings, we identified the curb mechanism between inclusive leadership and emotional exhaustion by merging the mediating role of caring ethical climate (organizational climate) and psychological safety (individual psychological characteristics). Consistent with our predictions the results of this study showed that inclusive leadership has a negative impact on employees’ emotional exhaustion. Caring ethical climate and psychological safety have a negative impact on employees’ emotional exhaustion and play a partially mediating role in the relationship between inclusive leadership and emotional exhaustion. Specifically, inclusive leadership curbed employees’ emotional exhaustion by reducing stress, creating a caring ethical climate, and filling the loss of employees’ psychological resources. Our study contributes to the literature on inclusive leadership and emotional exhaustion in several ways.

First, this study focused on the impact of inclusive leadership in the workplace on employees’ emotional exhaustion. The results extended the specific applications of inclusive leadership and COR theory to organizational behavior and enriched the existing literature on inclusive leadership and emotional exhaustion. Our study extended the effectiveness of inclusive leadership to emotional exhaustion in service settings. Inclusive leaders are more likely to pay attention to employees’ emotions and conflicts, position themselves as employees’ guiders, and tried to fill the loss of employees’ psychological resources, to alleviate the emotional exhaustion of employees.

Second, we found that caring ethical climate partially mediated the effect of inclusive leadership on employee emotional exhaustion. The results of this study showed that inclusive leadership has a direct impact on employees’ emotional exhaustion and had an indirect impact on employees’ emotional exhaustion by influencing their perception of caring ethical climate (Qi and Liu, 2017). It emphasized that the caring ethical climate is a booster of the effectiveness of inclusive leadership, believing that inclusive leadership can influence employees through the caring ethical climate. Emotional exhaustion caused by work stress is a resource-draining component of COR theory while a positive atmosphere of caring ethical climate, which helps to reduce employees’ negative behaviors and feelings, is a supplementary emotional resource, that consequently helps them recover from emotional draining.

Third, this study found that psychological safety mediates the relationship between inclusive leadership and emotional exhaustion. The indirect effect of inclusive leadership on emotional exhaustion through psychological safety is a further contribution to the literature on emotional exhaustion. In previous studies, inclusive leadership reduces employee deviant behavior by enhancing employee ownership (Ahmed et al., 2020; Lee and Dahinten, 2021). Fang et al. (2021) pointed out that psychological safety is one of the important variables to reveal the inclusive action mechanism and an important mediator between inclusive leadership and employee performance. Leadership influences emotional exhaustion through individual-level factors like intrinsic motivation and psychological empowerment (Tu and Lu, 2013; Afsar et al., 2014). Inclusive leadership to subordinates can improve the level of psychological safety of employees (Hirak et al., 2012), and stimulate the cognition and emotion of employees which in turn positively affects their ability and willingness (Zeng et al., 2020; Liu et al., 2021).

Fourth, this study constructed a dual mediation model of inclusive leadership, psychological safety, caring ethical climate, and emotional exhaustion. This study put forward inclusive leadership into the burnout syndrome frame, further confirming the inextricable relationship between leadership and emotional exhaustion. In advance studies, caring ethical climate and psychological safety are often used as antecedent variables of emotional exhaustion (Marchand and Vandenberghe, 2016) or subordinate variables of leadership (Zeng et al., 2020; Zhao et al., 2020). However, this study found that caring ethical climate and psychological safety mediate the relationship between inclusive leadership and emotional exhaustion. The finding of duo mediation effect of inclusive leadership on emotional exhaustion through caring ethical climate and psychological safety provided a more detailed mechanism for the inhibition of emotional exhaustion.

### Practical Significance

As a representative of the service industry, the hotel industry is an industry with more communication with customers. Moreover, the emotional exhaustion of hospitality front-line employees is typical. So that the exploration of inclusive leadership and emotional exhaustion can provide managers with prevention and coping strategies for burnout. The research results of this paper provided new ideas and perspectives for reducing emotional exhaustion. We found that it is very important to realize inclusive leadership, enhance the psychological safety of employees, create a caring ethical climate for the organization, and curb emotional exhaustion of employees under the long-term mechanism. These results have clear implications in hospitality organizations and are likely to be transferable to other service industries.

First, from an organizational perspective. Inclusive leadership can provide a harmonious, united, and caring ethical climate for the organization so that employees can perceive the support, attention, respect, and recognition from the organization. Our research found that inclusive leadership is an external driver for employees to avoid worries and reduce emotional exhaustion. Therefore, organizations should improve the leader selection process, increase the training of existing leaders, and strive to improve the inclusiveness of organizational leaders. Meanwhile, organizations should improve the focus and difficulty of leadership training by evaluating the leadership level of existing management teams to develop inclusive leaders.

Second, the organization should create an inclusive atmosphere through a series of measures such as work guidance and caring for employees, to improve their psychological safety of employees and stimulate their willingness to work. In addition, managers should strengthen emotional communication with employees as much as possible to truly reflect the “people-oriented” management concept. At the same time, the organization should create a relaxed and harmonious working atmosphere, to eliminate the depression and pressure on employees in their work. Employees also can eliminate the emotional exhaustion caused by work with the psychological support of the leader.

Finally, to reduce employee emotional exhaustion, our study suggested the value of actively paying attention to the caring ethical climate. Enterprises need to create a caring ethical climate to help employees achieve greater self-worth and stimulate their enthusiasm for work. At this time, employees can get the care and feel the warmth of the organization, to benefit their own emotions, such as creating a positive work mood and making physical and mental happiness. Leaders should also put more emphasis on positive interaction with employees, actively encourage and help employees to finish the tasks, enhance their sense of belonging, and strive to create a harmonious and friendly ethical climate.

### Research Limitations and Directions of Future Research

We also realized that there are many drawbacks to this study meanwhile we discussed the further direction of future research. Although our research has definite theoretical and practical significance, there are still some notable limitations. First, the collection of samples has limitations. Although we collected data from 65 companies in China, it is questionable whether the effects of inclusive leadership on emotional exhaustion can be similarly generalized to other samples due to the limited sample size and type of enterprises investigated in this study. To improve the generality, future studies in different industries may help complement the findings of this study.

Second, the results of this study explored and expanded the antecedents of emotional exhaustion. Before this, the antecedents of emotional exhaustion are extremely multiple, mainly including individual factors (emotion, cognition, personality traits, etc.) and organizational environmental factors (interpersonal relationship, working conditions, organizational structure, leadership, etc.). We hypothesized that individual factors and organizational environment may jointly influence emotional exhaustion. However, existing studies fail to combine the interaction between individual and organizational factors. Future studies should consider and combine the causes of different types or degrees of emotional exhaustion and comprehensively investigate how various influencing factors promote or relieve emotional exhaustion. For example, how to match leadership with employee traits to relieve emotional exhaustion or make leaders more effective.

Third, the results of this study suggested that the boundaries of inclusive leadership’s impact on emotional exhaustion need to be further explored. Based on COR theory, this study involved ethical climate and psychological safety to improve the influence mechanism of inclusive leadership on emotional exhaustion. Future research can further expand the theoretical research perspective of leadership behavior and emotional exhaustion by studying social exchange theory. At the same time, future research can be further carried out from the perspective of resource gain and loss.

## Conclusion

The results of this study confirmed that inclusive leadership can restrain employees’ emotional exhaustion in the workplace. This study provided a new theoretical basis and empirical research for the relationship between inclusive leadership and emotional exhaustion and helped us better understand the impact of inclusive leadership on emotional exhaustion.

In general, the contribution of this study is embodied in the following different aspects. First, based on COR theory, this paper established a dual intermediary theoretical model of inclusive leadership, psychological safety, caring ethical climate, and emotional exhaustion. Our findings suggested that inclusive leadership has a negative impact on employee emotional exhaustion in Chinese organizational Settings. Second, our results demonstrated that caring ethical climate and psychological safety partially mediate the relationship between inclusive leadership and emotional exhaustion. This study also added to our understanding of the relationship between inclusive leadership and employee behavior. Thirdly, the potential research value of this paper is to encourage more organizations or enterprises to adopt inclusive leadership in current business operations, to effectively suppress emotional exhaustion, improve organizational adaptability and innovation ability, and enhance employees’ job satisfaction and personal growth potential.

## Data Availability Statement

The raw data supporting the conclusions of this article will be made available by the authors, without undue reservation.

## Author Contributions

PP contributed to conception and design of the study. XL organized the database and performed the statistical analysis. PP and XL wrote the first draft of the manuscript. All authors contributed to the article and approved the submitted version.

## Funding

This work was supported by Shandong Jianzhu University Ph.D. Fund Project (XNBS643).

## Conflict of Interest

The authors declare that the research was conducted in the absence of any commercial or financial relationships that could be construed as a potential conflict of interest.

## Publisher’s Note

All claims expressed in this article are solely those of the authors and do not necessarily represent those of their affiliated organizations, or those of the publisher, the editors and the reviewers. Any product that may be evaluated in this article, or claim that may be made by its manufacturer, is not guaranteed or endorsed by the publisher.
